# From Horror to Hope: Recognizing and Preventing the Health Impacts of War

**DOI:** 10.5334/aogh.3860

**Published:** 2022-08-18

**Authors:** Michael S. Baker

**Affiliations:** 1 Rear Admiral, Medical Corps, USN (ret), US

**Keywords:** Book review, war casualties, PTSD, mental health, impacts of war

*From Horror to Hope: Recognizing and Preventing the Health Impacts of War*, by Dr. Barry Levy is an excellent and scholarly contribution to the understanding of the extensive and pervasive negative impacts of war. This topic is more relevant than ever, because now – in contrast to most wars in the last 75 years that transpired far from those of us living in western nations – we have an unjust conflict imposed by Russia on Ukraine bringing it into our collective consciousness.

This is a heartbreaking book to read because of the scope and severity of human suffering that we inflict on each other. It enhances ones’ understanding of the health impacts and costs of war, from smaller confined intrastate conflicts in faraway lands to the astronomical casualty rates of global conflict in World War II. Given the variety and location of conflicts, and the difficulties of acquiring meaningful data from conflict zones, the presentation is well done, and the work is well annotated. Dr. Levy also documents the interconnectedness and relationship of the various negative impacts of war and conflict and draws a line from war to impaired health, chronic illness, early death, as well as future societal problems.

Dr Levy clearly points out that war causes not only casualties, deaths, and spreads diseases, but also affects mental health of both the wounded victim and the witness. War forcibly displaces people, damages essential elements of civilian infrastructure, contaminates the environment, and diverts resources to military uses from health, education, and other services of societal well-being. And worst of all, it often begets more violence. He notes that the risks for future wars appear to be on the rise due to increasing nationalism and populism in many countries, growing availability of lethal weapons, mass displacement of people accompanied by political and socioeconomic instability, and the increasing disruptions of climate change. He calls out the militarism in speech and actions that normalizes war and preparation for war by influencing mass culture and consciousness, politics, and economic priorities.

Dr. Levy writes his book in the hope that we all recognize the staggering negative impacts of war and work to prevent them. He is optimistic with regards to the value of pursuing diplomacy, the stability value of international norms and conventions, and the promise of international organizations such as the United Nations. He highlights certain of these conventions such as the Convention on the Rights of the Child (CRC), and the International Covenant on Civil and Political Rights (ICCPR), and the UN Convention Against Torture and other Cruel, Inhuman or Degrading Treatment or Punishment (UNCAT). His prescription for change includes participating in and promoting civil society organizations that can identify and investigate disputes and help resolve them nonviolently. These organizations and their members can help de-escalate violence, improve communication, and further political stability.

Other recommendations to prevent war and its painful sequela include improving citizen participation in community affairs, reinforcing democratic government through free elections, and defending other democratic processes such as free speech and press – which makes government more accountable and decreases the “forgotten” individuals’ sense of grievance. Mechanisms to promote the rule of law and to ensure justice are essential to good governance. Strengthening civil society organizations also supports citizen participation and government responsiveness. Improving governance helps to reduce the likelihood of civil conflict and war. These are tools we may soon need in this country, where the trouble cauldron appears to be simmering and ready to boil over.

He does not ignore nor dismiss the actions of the United States, which he notes is not a signatory to many of the international conventions. He calls out the U.S. for perhaps launching unjustifiable wars by invading Iraq and Afghanistan He notes the U.S. military budget dwarfs the next ten countries military budgets combined while being a massive contributor to pollution and global climate change. Again, his point is that more weapons and more militarism in speech and actions can result in more wars.

The book is well seasoned with excellent quotes – from intellectual luminaries such as Einstein, Kierkegaard, the Dali Lama, and Sartre – that fit well into the topics presented. The highlight for me as a reader was the inclusion of profiles of numerous heroes of global public health who, through their commitment in giving of themselves while helping others, exemplify the best of our humanity. Their stories are quite inspiring and show that each of us has a role that we can fulfill in making the world safer for our children and grandchildren.

